# Comparing Learning Outcomes of Virtual Reality (VR) Simulators Using Haptic Feedback Versus Box Trainer (BT) in Laparoscopic Training: A Systematic Review and Meta-Analysis

**DOI:** 10.7759/cureus.78910

**Published:** 2025-02-12

**Authors:** Janice Tan, Md Rezaul Karim, Rezuana Tamanna, Sola Kim, Bijendra Patel

**Affiliations:** 1 Surgery, Barts Cancer Institute, London, GBR; 2 Surgery, The Royal London Hospital, London, GBR; 3 General Surgery, Watford General Hospital, Watford, GBR; 4 Surgical Sciences, Barts Cancer Institute, London, GBR

**Keywords:** box trainer, haptic feedback, laparoscopic training, medical students, surgical simulation, virtual reality

## Abstract

Minimally invasive laparoscopic surgery requires intensive training due to challenges such as loss of haptic feedback and depth perception. Traditional training methods include box trainers (BT), which offer realistic haptic feedback but lack objective performance assessment, and virtual reality (VR) simulators, which provide automated feedback but lack haptic feedback. This review, conducted at the Barts Cancer Institute, Queen Mary University, examines the learning outcomes of VR simulators with haptic feedback compared to BT.

A systematic review and meta-analysis was conducted following Preferred Reporting Items for Systematic Reviews and Meta-Analyses (PRISMA) guidelines from December 2023 to April 2024. Research databases, such as PubMed, EMBASE, CINAHL, and Web of Science, were searched for randomized controlled trials (RCTs) comparing VR simulators with haptic feedback to BT in training medical students. Seven RCTs met the inclusion criteria, and four were included in the meta-analysis. The primary outcomes were learning curves and learning effects while secondary outcomes included skill transfer to a surgical environment. The review analysed data from 125 participants across the studies. Results indicated that BTs demonstrated a superior learning curve, with participants achieving proficiency faster than those using VR. Both simulators showed significant learning effects; however, BTs resulted in greater improvements across more performance parameters. Regarding skill transfer to surgical environments, there was no significant difference between the two groups, suggesting both approaches effectively support surgical skill transfer. Overall, BT has a more effective learning curve and marginally better performance in skill acquisition. While VR with haptic feedback offers enhanced realism, it does not fully replicate the natural haptic feedback provided by BT. Further studies are needed to improve VR haptic feedback and its integration into training programs to enhance learning outcomes.

## Introduction and background

Haptics, defined as recognising objects through touch via force feedback, are crucial in laparoscopy simulation training [1]. The current surgical simulation systems include box trainers (BT) and virtual reality (VR) simulators. BT provides realistic haptic feedback but lacks objective assessment, requiring an observer to be present [2,3]. The Fundamentals of Laparoscopic Surgery (FLS) box trainer is currently the standard for assessing laparoscopic surgical skills [4]. BT are advantageous because they are mobile, cost-effective, and allow practice on animal tissue, enhancing procedural realism [5]. In contrast, VR simulators offer objective assessment and facilitate performance monitoring [2,3]. The advantages of VR simulators are their reusability without consumables and their automated feedback for efficient training. However, most VR simulators lack haptic feedback [4]. Examples include the Minimally Invasive Surgery Trainer - Virtual Reality (MIST-VR; Mentice, Gothenburg, Sweden) [6], SIMENDO (Simendo B.V., Rotterdam, The Netherlands) [7], and SimSurgery (SimSurgery AS, Oslo, Norway) [8].

This led to the development of high-fidelity laparoscopic VR stimulators which include haptic feedback but come at a higher cost [1]. Examples include LAP Mentor (Simbionix, Cleveland, OH, USA), LapSim (Surgical Science, Gothenburg, Sweden) [8], and Virtual Basic Laparoscopic Skill Trainer (VBLaST; Rensselaer Polytechnic Institute, Troy, New York, USA) [9]. It is shown that haptic feedback enhances the realism and training effect of VR simulators [1]. However, the degree of realism varies depending on the software programme, thus VR simulators can only offer a part of physical reality. Hence, it remains in question whether skills acquired in VR simulators can be transferred to the surgical environment [10] and whether VR simulators can replicate the natural haptic feedback of BT [5].

Lastly, it is suggested that novice medical students may benefit most from simulation training, showing faster improvement than experienced surgeons, who often have a steeper learning curve. This may be due to the increased age, which can worsen simulator skills, highlighting the importance of early training [11]. Alternatively, experienced surgeons might adapt more quickly to the simulator, thus resulting in a shorter learning curve. Hence, individuals with less laparoscopic experience might benefit more compared to those with more experience [12].

Literature review

The current literature suggests that while VR simulators come with significant advantages as a training tool, they show no significant difference in learning outcomes as compared to BT. Rangarajan et al. demonstrated that VR simulators with haptic feedback improve training efficacy compared to VR simulators without haptics [1]. In contrast, Jin et al. showed no significant difference between the learning curves of VR simulators and BTs, suggesting that VR simulators are not recommended as a training tool for laparoscopic surgery [11]. Similarly, Overtoom et al. showed inconclusive results in VR simulators with haptic feedback but positive outcomes with BTs [13]. However, a limitation of these studies is that the haptic features of the VR simulators were not adequately addressed.

Study aims

To date, there are no reviews comparing the learning outcomes of VR simulators with haptic feedback to BTs. Therefore, this review primarily seeks to investigate whether the incorporation of haptic feedback in VR simulators leads to significant differences in learning outcomes compared to BTs. This is measured by the learning curve, which tracks participants' performance improvement over time, and the learning effect, which measures the immediate performance gains from pre-training to post-training test scores. The secondary aim is to determine whether VR simulators with haptic feedback result in better evidence of skill transfer to surgical models compared to BTs. This refers to the ability to apply skills acquired through simulator training to mimic real surgeries.

This article was previously presented as a free paper presentation at the 2024 ALSGBI Annual Scientific Meeting on November 6, 2024.

## Review

Material and methods

Study Design

This systemic review and meta-analysis aimed to compare the learning outcome between VR simulators with haptic feedback and BT. This review was conducted at Barts Cancer Institute, Queen Mary University of London, from December 2023 to April 2024, following the PRISMA guidelines. The protocol was registered with the Prospective Register of Systematic Review (PROSPERO) on 28 March 2024, under the registration number CRD42024529489 (available at https://www.crd.york.ac.uk/PROSPERO/display_record.php?RecordID=529489).

The target population for this review is medical students. Medical students were chosen because they are considered true novices in laparoscopic training, having the least experience in the field and the greatest potential for improvement. The primary outcome, the learning curve is measured using two key metrics: the 5% acceptable failure rate, which is the threshold where participants fail no more than 5% of the time, and the transition point, the moment when participants achieve consistent performance improvement. The learning effect is measured by comparing pre and post-training test scores. These were chosen as the primary outcomes because they are widely reported in the literature and are believed to provide an objective comparison of the effectiveness of VR simulators and BTs. The secondary outcome, skill transfer to a surgical model, is assessed by comparing the performance of VR simulators and BTs to ex-vivo models.

Search Strategy

The search was conducted using the following databases: PubMed, EMBASE, Web of Science, and Cochrane. The following search terms were included: ("student" OR "novice" OR "junior") AND ("Virtual reality" OR "VBLaST" OR "haptic" OR "force feedback" or "tactile feedback" OR "high fidelity" OR “lapsim” OR “lapmentor” OR “simbionix”) AND ("Fundamentals of Laparoscopic Surgery" OR "BT simulator" OR "box trainer" OR "box training") AND ("Laparoscop*" OR "surgical training" OR "skill training" OR "minimally invasive" OR "laparoscopy/education"[MeSH Terms] OR "simulator"). All selected papers were stored in EndNote for referencing and duplicate removal. Additionally, the reference lists of relevant articles and grey literature were manually reviewed. Outcomes were not included in the search strategy to maximise the number of papers screened. This review included papers published from 2000 onwards and the date of the last search was 8 February 2024. The year 2000 onwards was chosen because this period marks the introduction of laparoscopic training, making it a more relevant timeframe for assessing modern training methods.

Inclusion and Inclusion Criteria

Table 1 lists the criteria used to include or exclude study participants.

**Table 1:** Inclusion and exclusion criteria VR: virtual reality; BT: box-trainers; RCTs: randomised controlled trials; NRCTs: non-randomised controlled trials

	Include	Exclude
Population	Medical student (Year 1 – 5)	Surgical trainees, medical registrars, surgeons, or experience with laparoscopic simulation
Intervention	VR simulator with haptic (i.e LAP Mentor, LapSim, VBLaST) or as stated otherwise	VR simulators without haptic feedback, or other non-VR simulators such as robotics, augmented reality, video trainers
Comparator	Box Trainer or pelvic trainer	Other simulators such as robotic, augmented reality, video trainer
Study Design	Comparative studies on VR simulators and BT, RCTs, published in the English literature, published after 2000, available through Queen Mary University of London's library	Case studies, case reports, study proposals, systemic reviews, conference abstracts, NRCTs

Data Extraction

Two review authors independently conducted the screening process. Any disagreements were resolved by discussion and asking another author if needed. Data extracted from the included articles included the name of the author, published year, title of study, type of study, participants, types of VR, task, outcome measurement and result findings. All systematically extracted data were synthesised using Excel and Word documents (Microsoft Corporation, Redmond, WA, US).

Methodological Quality

The RoB-2 tool (Cochrane Collaboration, London, UK) was used to assess the risk of bias in randomised controlled trials (RCTs). This tool evaluates the following elements: randomisation process, deviations from the intended intervention, missing outcome data, measurement of the outcome and selection of the reported result. The GRADE (Grading of Recommendations, Assessment, Development, and Evaluations) guideline was used to evaluate the level of evidence using the GRADEpro Guideline Development Tool software by Cochrane. This assessed the following factors: risk of bias, inconsistency, imprecision, indirectness and publication bias.

Statistical Analysis

Revman 5.3 software was used to present the data in the meta-analysis. Risk ratio and 95% confidence intervals were calculated for dichotomous variables, using the random effect model with the inverse variance method. Results were illustrated in forest plots, with a *p*-value ≤ 0.05 considered statistically significant. Heterogeneity was assessed using the I² statistic, where an I² value of less than 25% indicates low heterogeneity, I² between 25% and 50% indicates moderate heterogeneity, and I² greater than 50% indicates high heterogeneity.

Results

Description of Studies

**Figure 1:**
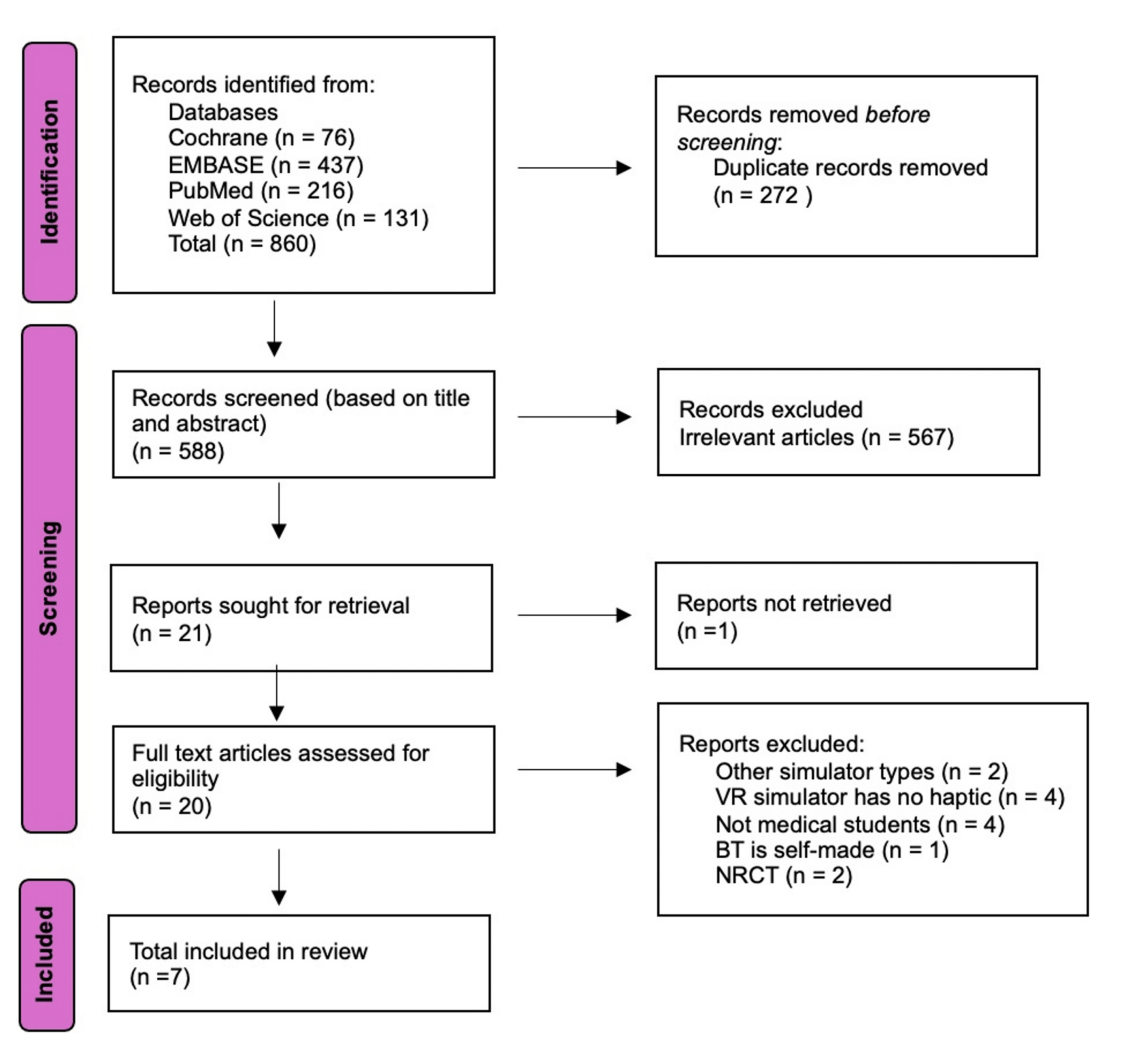
PRISMA flowchart PRISMA: Preferred Reporting Items for Systematic Reviews and Meta-Analyses; VR: virtual reality; BT: box trainer; NRCT: non-randomised controlled trial

**Figure 2:**
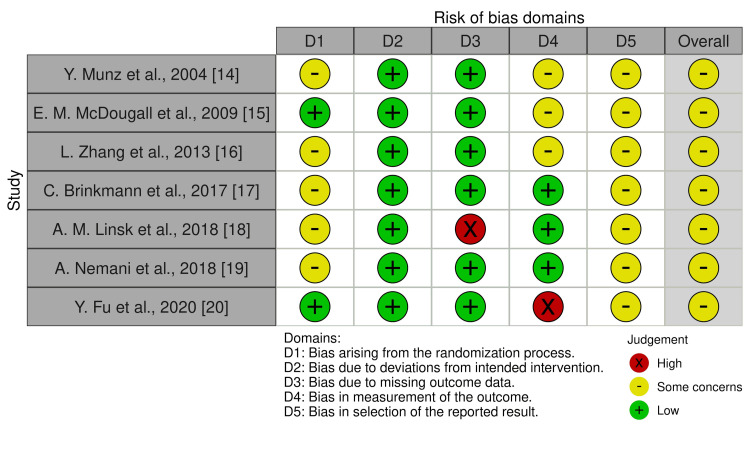
The RoB-2 summary of all included studies RoB 2: Revised Cochrane Risk-of-Bias tool for randomised trials References: [14-20]

A total of seven studies, published between 2004 and 2020, were included in this systematic review, as illustrated in the Preferred Reporting Items for Systematic Reviews and Meta-Analyses (PRISMA) flow diagram (Figure 1). These RCTs involved 125 medical students, with 62 in the VR group and 63 in the BT group. The study characteristics of the included studies are shown in Table 2. The primary outcomes measured in this review are the learning curve and learning effect. The secondary outcome is skill transfer to the surgical model. The RoB-2 tool used to assess the risk of bias is illustrated in Figure 2.

**Table 2:** Summary of study characteristics VR: virtual reality; BT: box trainer References: [14-20]

Study	Number of participants			VR simulator model	Method of assessment	Outcomes measured	Risk of bias
	Total	VR	BT				
Y. Munz et al., 2004 [14]	16	8	8	LapSim	Laparoscopic task: manipulation of the model, grasping, cutting, and clip-applying	Learning effect	Some concern
E. M. McDougall et al., 2009 [15]	20	10	10 (pelvic trainer)	LAP Mentor	The final assessment is laparoscopic cystorrhaphy (suturing and knot tying) in the porcine model	Skill transfer to surgical model	Low
L. Zhang et al., 2013 [16]	12	6	6	VBLaST	Peg transfer	Learning curve, learning effect	Some concern
C. Brinkmann et al., 2017 [17]	36	18	18	LAP Mentor	Peg transfer and Pattern Cutting. Skill transfer is measured by assessment of ex situ laparoscopic cholecystectomy on pig liver in BT.	Learning effect, skill transfer to surgical model	Low
A. M. Linsk et al., 2018 [18]	14	7	7	VBLaST	Pattern cutting	Learning curve, learning effect	Some concern
A. Nemani et al., 2018 [19]	13	6	7	VBLaST	Pattern cutting and skill transfer are measured by PC on an ex vivo tissue model.	Learning curve, skill transfer to the surgical environment	Low
Y. Fu et al., 2020 [20]	14	7	7	VBLaST	Intracorporeal suturing	Learning effect, learning curve	Some concern

Descriptive Statistics of the Learning Curve

The learning curve was studied in four papers, as listed in Table 3. This was assessed by the number of participants that achieved the 5% acceptable failure rate and met the transition point. The level of performance for these two was broken down into junior, intermediate, and senior levels. There was a total of 53 participants split into the VR simulator group (26 participants) and the BT group (27 participants). The mean number and percentage of participants to reach the 5% acceptable failure rate is higher in the BT group (4.86, 54%) than in the VR simulator group (2.43, 39.4%). The mean number and percentage of participants that reach the transition point is higher in the BT group (6.17, 95.3%) as compared to the VR simulator group (5.5, 85.8%).

**Table 3:** Learning curve in VR simulator and BT VR: virtual reality; BT: box trainer References: [16,18-20]

		VR simulator				BT			
Study	Task	Number of participants	Level of performance (level/score)	Number of participants that reach the 5% acceptable failure rate (%)	Number of participants that reach the transition point (%)	Number of participants	Level of performance (level/score)	Number of participants that reach the 5% acceptable failure rate (%)	Number of participants that reach the transition point (%)
L. Zhang et al., 2013 [16]	Peg Transfer	6	Junior score/ 59	5 (83%)	6 (100%)	6	Junior score/ 41	6 (100%)	6 (100%)
			Intermediate score/ 63	3 (50%)	6 (100%)		Intermediate score/ 65	6 (100%)	6 (100%)
			Senior score/ 68	2 (33%)	6 (100%)		Senior score/ 78	3 (50%)	6 (100%)
A.M. Linsk et al., 2018 [18]	Pattern Cutting	7	Intermediate score/ 52	2 (29%)	7 (100%)	7	Intermediate score/ 56	5 (71%)	7 (100%)
			Senior score/ 72	0 (0%)	2 (29%)		Senior score/ 72	0 (0%)	6 (86%)
A. Nemani et al., 2018 [19]	Pattern Cutting	6	Senior score/ 63	4 (67%)	N/A	7	Senior score/ 63	3 (43%)	N/A
Y. Fu et al., 2020 [20]	Intracorporeal suturing	7	Intermediate score/ 280.8	1 (14%)	6 (86%)	7	Intermediate score/ 280.8	1 (14%)	6 (86%)
		Total = 26		Mean = 2.43 (39.4%)	Mean = 5.5 (85.8%)	Total = 27		Mean = 4.86 (54.0%)	Mean = 6.17 (95.3%)

Meta-analysis of the Learning Curve 

**Figure 3:**
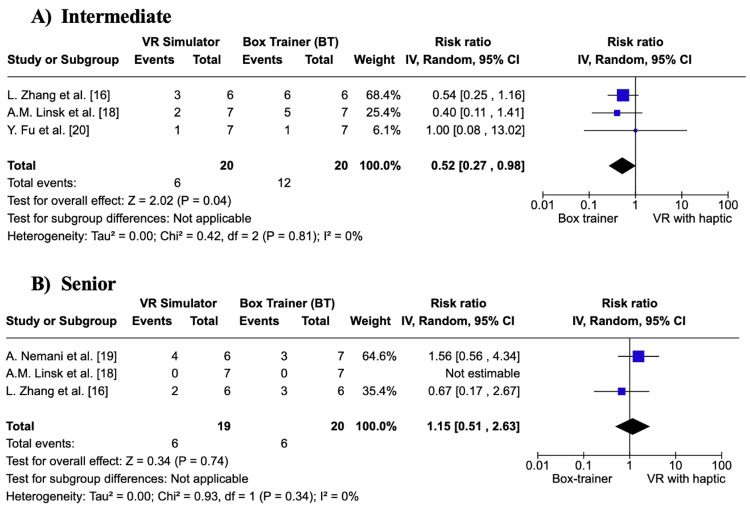
Forest plot of the number of participants that reached the 5% acceptable failure rate (%): A) Intermediate B) Senior Meta-analysis with inverse variance random effects model VR: virtual reality; IV: inverse variance; CI: confidence interval References: [16,18-20]

In Figure 3A, the BT group had a significantly greater number of participants that reached the 5% acceptable failure rate at the intermediate level as compared to the VR simulator group (p = 0.04). In Figure 3B, the BT group and VR simulator group had no significant difference in the number of participants that reached the 5% acceptable failure rate at the senior level (p = 0.74). In Figures 3A, 3B, no statistical heterogeneity was observed (I² = 0).

**Figure 4:**
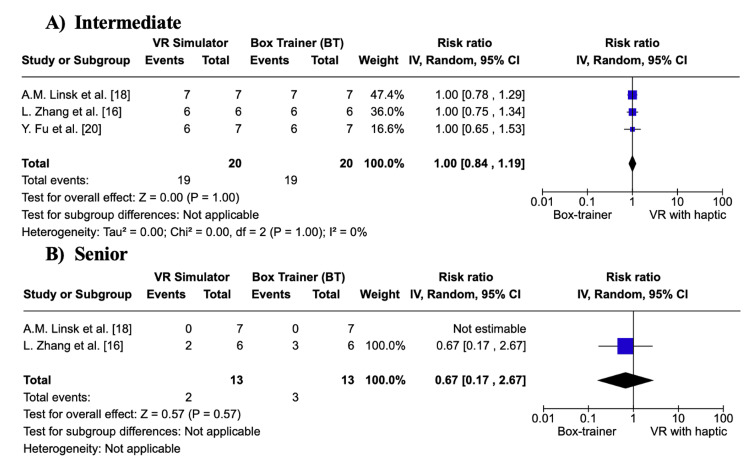
Forest plot of the number of participants that reached the 5% transition point (%): A) Intermediate B) Senior Meta-analysis with the inverse variance random effects model VR: virtual reality; IV: inverse variance; CI: confidence interval References: [16,18,20]

In Figure 4A and Figure 4B, the BT group and VR simulator group had no significant difference in the number of participants that reached the transition point at both the intermediate and senior levels (p = 1.00; p = 0.57). In Figure 4A, no statistical heterogeneity was observed (I² = 0). 

Overall, the results show that both BT and VR simulators are effective tools for laparoscopic surgical training. In terms of overall skill acquisition at all levels, more participants reach the 5% acceptable failure rate in BTs. At the intermediate level, significantly more participants in the BT group achieved the 5% failure rate. However, at the senior level, there is no significant difference in skill acquisition between BTs and VR simulators.

Descriptive Statistics of the Learning Effect

The learning effect was studied in four papers, as listed in Table 4. There was a total of 76 participants split into the VR simulator group (38 participants) and the BT group (38 participants). The VR simulator group reports a significant difference in pre-post test scores in 9 out of 12 data points; the BT group reports a significant difference in pre-post test scores in 11 out of 12 data points. Overall, both simulators demonstrate significant learning effects, with the BT group showing significant improvement in more parameters compared to the VR simulator.

**Table 4:** Learning effect in the VR simulator and box trainer *Note:* Raw data was not provided in the included studies. * Indicates *p*>0.05 VR: virtual reality; BT: box trainer References: [14,16,17,20]

			VR simulator		BT	
Study	Task	Outcome measurement	Number of participants	Comparison of Pre-test and Post-test (p-value)	Number of participants	Comparison of Pre-test and Post-test (p-value)
Y. Munz et al., 2004 [14]	Laparoscopic task: manipulation of the model, grasping, cutting, and clip-applying	Total number of movements	7	0.836*	7	0.012
		Total path length by both hands		0.088*		0.012
		Time taken for completion		0.123*		0.123*
		Economy of movement		0.05		0.036
		Distance travelled		0.05		0.012
		Number of errors		0.017		0.012
L. Zhang et al., 2013 [16]	Peg Transfer	Performance score	6	< 0.002	6	0.001
C.Brinkmann et al., 2017 [17]	Peg Transfer	Task time	18	< 0.001	18	< 0.001
	Pattern Cutting			< 0.001		< 0.001
	Ligating Loop			0.001		< 0.001
	Knot Tying			< 0.001		< 0.001
Y. Fu et al., 2020 [20]	Intracorporeal Suturing	Performance score	7	< 0.001	7	< 0.001

Descriptive Statistics of Skill Transfer to the Surgical Model

Skill transfer to the surgical model was studied in three papers, as shown in Table 5. There was a total of 69 participants, split into the VR simulator group (34 participants) and the BT group (35 participants), who only practised with their respective simulators. Two out of three studies reported no significant difference in results between the BT and VR simulator groups. Overall, there is no significant difference in performance scores between simulators when evaluating skill transfer to the surgical model. 

**Table 5:** Skill transfer to the surgical model in VR simulator and BT * Indicate *p*>0.05 VR: virtual reality; BT: box trainer; SE: standard error; OSATS: Objective Structured Assessment of Technical Skill; GOALS: Global Operative Assessment of Laparoscopic Skills References: [15,17,19]

			VR simulator		BT group		
Study	Surgical Model	Method of assessment	Number of participants	Mean ± SE	Number of participants	Mean ± SE	P- value
E. M. McDougall et al., 2009 [15]	Laparoscopic cystorrhaphy (suturing and knot tying) in porcine model	OSATS score (out of 25)	10	8.2 ± 2.2	10	8.8 ± 2.3	0.24*
		Time taken (mins)		41 ± 10		40 ± 15	0.87*
C.Brinkmann et al., 2017 [17]	Ex-situ laparoscopic cholecystectomy	GOALS score (sum of items 1-5)	18	15.31 ± 3.61	18	12.92 ± 3.06	0.039
		Depth Perception		2.67 ± 0.66		3.31 ± 0.84	0.016
		Efficiency		2.42 ± 0.83		3.06 ± 0.94	0.037
Nemani et al., 2018 [19]	Pattern cutting on ex vivo cadaveric models	Mean time completion (mins)	6	7.9 ± 3.3	7	12.3 ± 1.9	> 0.05*

Summary of Findings

Table 6 summarises the findings of this study.

**Table 6:** Summary of findings for all outcomes measured in the included RCTs Population – Medical students Intervention – VR simulator with haptic Comparator – Box trainer (BT) Outcomes – Learning curve, learning effect, skills transfer to surgical model Study design – Systemic review and meta-analysis GRADE Working Group grades of evidence: High Certainty: We are very confident that the true effect lies close to that of the estimate of the effect. Moderate Certainty: We are moderately confident in the effect estimate; the true effect is likely to be close to the estimate of the effect, but there is a possibility that it is substantially different. Low Certainty: Our confidence in the effect estimate is limited; the true effect may be substantially different from the estimate of the effect. Very Low Certainty: We have very little confidence in the effect estimate; the true effect is likely to be substantially different from the estimate of the effect. a. Different tasks were carried out between studies and the level of performance score varies between studies. b. Different tasks were carried out with different outcome measurements. c. Two studies reported no significant difference in performance scores between VR and BT. One study reported a significant difference d. Different "surgical models" were used across studies. e. Different tasks and outcome measurements are used. CI: confidence interval; VR: virtual reality; BT: box trainer

Outcomes	Data recorded	% of papers reporting (Total = 7)	Number of participants		Impact	Quality of evidence (GRADE)	Comment
			VR Simulator	BT			
Learning curve (assessed with: Number of participants reach the 5% acceptable failure rate)	YES – 4 NO – 3	50%	17/45 (37.8%)	24/46 (52.2%)	Junior: Insufficient studies to conduct meta-analysis; Intermediate: Risk ratio: 0.52, 95% CI 0.27–0.98, p = 0.04; Senior: Risk ratio: 1.15, 95% CI 0.51–2.63, p= 0.74	⨁⨁◯◯ Low ^a^	RCTs with some concern were included. High certainty of evidence but different tasks between studies affect the precision.
Learning curve (assessed with: Number of participants that reach the transition point)	YES – 4 NO – 3	50%	33/45 (73.3%)	37/46 (80.4%)	Junior: Insufficient studies to conduct meta-analysis; Intermediate: Risk ratio: 1.00, 95% CI 0.84–1.19, p = 1.00; Senior: Risk ratio: 0.67, 95% CI 0.17–2.67, p= 0.57	⨁⨁◯◯ Low ^a^	RCTs with some concern were included. High certainty of evidence but different tasks between studies affect the precision.
Learning effect (assessed with: Comparison of Pre-test and Post-test (p-value))	YES – 4 NO – 3	50%	38	38	Four out of seven papers reported outcomes on comparison of pre-test and post-test. No statistical analyses can be done as only the p-value is provided. Overall, the VR simulator reported a significant difference in 9/12 data points and the BT simulator reported a significant difference in 11/12 data points.	⨁⨁◯◯ Low ^b^	RCTs with some concern were included. High certainty of evidence but different tasks and assessment methods between studies affect the precision.
Skill transfer to surgical model	YES – 3 NO – 4	38%	34	35	Three out of seven papers reported outcomes on skill transfer to the surgical model. No statistical analyses can be done, as different parameters were assessed across studies leading to heterogeneity of data.	⨁◯◯◯ Very low ^c,d,e^	Low certainty of evidence due to inconclusive findings and variation in tasks and methods of assessment.

Discussion

The overall findings demonstrate that BT has a significantly better learning curve compared to VR simulators. This is shown by more participants reaching the 5% acceptable failure rate and meeting the transition point in the BT group. Regarding the learning effect, the BT group showed significant improvement in more parameters compared to the VR simulator group. Lastly, there is no significant difference in performance scores between simulators when evaluating skill transfer to the surgical model.

Primary Outcome: Learning Curve

Learning curves measure gains in performance with repetition and have been widely used in assessing surgical training. They typically show rapid initial improvements that plateau as proficiency is approached [16]. Results from three out of four studies showed that BTs led to better skill acquisition compared to VR simulators [17,18,20]. Only Nemani et al. reported comparable performance between the two groups, with similar numbers of participants achieving the “senior” level, suggesting both simulators are equally effective for complex tasks [19]. This supports results from the meta-analysis, which suggests that BTs outperform VR simulators in the early training stages only. Findings for the number of participants reaching the 5% acceptable failure rate showed a significant difference at the intermediate level (p = 0.04) but not at the senior level (p = 0.74). This suggests that both simulators are equally effective once higher proficiency is attained. Similarly, no significant differences were found in the number of participants reaching the transition point at either skill level, highlighting a ceiling effect where simulator type becomes less influential.

Outcomes are shown to vary by task difficulty. For example, in Linsk et al.’s study, no participants achieved the senior threshold score in pattern cutting, likely due to its complexity, whereas peg transfer showed better results. However, the BT group reached the transition point earlier, possibly due to faster familiarisation [18]. Similarly, intracorporeal suturing, a challenging laparoscopic task, had fewer participants achieving the 5% failure rate in both simulator groups, though this is expected to improve with additional practice [20]. Overall, these findings suggest that while BTs have an advantage in the early stages of training, both simulators are equally effective in achieving advanced skill levels.

Primary Outcome: Learning Effect

The learning effect is demonstrated by the impact of repetitive training on performance. Overall, both the BT and VR simulator groups demonstrate significant improvement in pre- to post-test scores, with the BT group showing significant improvement in more parameters compared to the VR simulator group. Results from the included studies showed significant improvements within their respective simulators, highlighting the specificity of training environments [14,16,17,20]. Various tasks were evaluated (e.g., peg transfer, pattern cutting, ligating loop, knot tying, suturing), supporting the effectiveness of both simulators as training modalities.

Interestingly, Y. Munz et al. found no significant improvement in task completion time for either simulator. This could be due to participants prioritising accuracy over speed as proficiency increased. Additionally, only the VR simulators showed no significant improvement in total movements or path length, likely due to a longer adaptation period to VR technology. However, these outcomes could potentially improve with increased practice [14].

Secondary Outcome: Skill Transfer to the Surgical Model

Both E. M. McDougall et al. and Nemani et al. found no significant differences in the transfer task completion time between the VR simulator and BT groups [15,19]. Regardless, skill transfer is evident in both simulators. Nemani demonstrated that both the BT group (7.9 ± 3.3) and the VR simulator group (12.3 ± 1.9) completed the transfer task significantly faster than the control group (18.4 ± 3.1, p < 0.05) [19]. Furthermore, E. M. McDougall et al. showed that inexperienced medical students were able to perform intracorporeal suturing, one of the most challenging laparoscopic tasks, on a porcine model within two to three hours of training [15].

However, only Brinkmann et al. reported a more significant skill transfer with the BT group compared to the VR simulator group. It was suggested that prior experience with BTs might positively influence cholecystectomy performance in the box trainer. Notably, there is a recognisable gap between simulator training and real operations, but this gap appears narrower with BTs, which closely mimic actual procedures. BTs use actual surgical instruments, offering real haptic feedback and making skills more transferable to real-world conditions [17].

Limitations

This review has several limitations. Firstly, it only included medical students, which may affect the generalisability of the findings. Medical students were selected because they are considered true "novices" in surgery, with minimal prior experience. However, laparoscopic training is intended for all surgeons, and a broader demographic should be considered to improve the applicability of the results.

Another limitation is the variation in outcome measures and task difficulty across the included studies. Different methods were used to assess performance, and the difficulty levels of the tasks varied across studies. These inconsistencies introduced significant heterogeneity, making data synthesis for meta-analysis challenging and potentially affecting the precision of the results. This review attempts to mitigate this by conducting a meta-analysis separately for different performance levels (intermediate and senior). Besides this, the limited number of studies further complicates the validity of the meta-analysis, and results should therefore be interpreted with caution. This is evident in the I² score of 0 observed in this review, which, despite indicating no statistical heterogeneity, is based on only three studies; hence, methodological heterogeneity cannot be ruled out.

This review highlights the need for more uniform methodological standards, including consistent outcome measures and the development of standardised assessment tools. Addressing these issues would enhance comparability and improve understanding of the effectiveness of simulation in surgical training.

## Conclusions

In conclusion, BTs offer a more engaging learning experience, resulting in a steeper learning curve compared to VR simulators. They have been shown to outperform VR simulators in the early training stages and facilitate greater skill acquisition across multiple parameters, making them the preferred choice for laparoscopic training. In contrast, the effectiveness of VR simulators is currently constrained by technological limitations, particularly in haptic feedback, which has yet to replicate the natural tactile sensations provided by BTs.
